# A three day coaching protocol for deep inspiration breath hold in left breast radiotherapy using active breathing control

**DOI:** 10.1038/s41598-025-30484-6

**Published:** 2025-12-04

**Authors:** Siqi Jiang, Haokun Shi, Xi Ye, Jun Yang, Jian Zheng, Peiwei Wu, Qiang Ye, Shuanglai Qin, Wenjie Liu, Zuowei Hu, Yu Cai

**Affiliations:** Department of Radiation Oncology, Wuhan No.1 Hospital, Wuhan, Hubei China

**Keywords:** Left-side breast radiotherapy, Respiratory maneuver, Deep inspiration breath-hold, Active breathing control system, Breath coaching, Breath-hold threshold, Breast cancer, Radiotherapy

## Abstract

The deep inspiration breath-hold (DIBH) technique is recommended for left-sided breast radiotherapy to reduce heart dose exposure. However, this technique introduces uncertainties due to possible anatomical variations across successive breath-holds, requiring patient coaching to ensure reproducibility. This study aims to establish an objective coaching framework to optimize DIBH effectiveness and achieve the reproducibility of chest anatomy in left-sided breast radiotherapy. This study implemented a three-day DIBH coaching protocol using the Active Breathing Coordinator (ABC) system (Elekta AB, Sweden), with particular emphasis on the breath-hold threshold and the lung volume difference from CT images. The former was a manually configured parameter in the ABC system that reflected the stability of patient’s respiratory maneuvers during coaching. The latter was used to quantify the DIBH reproducibility. Quantitative analysis demonstrated that for the 9 patients who exhibited fluctuations in their breath-hold threshold, continued coaching successfully enabled them to achieve stability. Another 14 patients maintained a consistent breath-hold threshold throughout the entire coaching process. Only one patient showed deterioration in this parameter. Additionally, the breath-hold threshold showed significant positive correlations with DIBH lung volumes: left lung (*r* = 0.638, *p* = 8.028 × 10^−4^), right lung (*r* = 0.647, *p* = 6.284 × 10^−4^). Regarding reproducibility of DIBH, 78.3% (18/23) and 73.9% (17/23) of patients maintained ≤ 0.1 L difference between two DIBH scans for left and right lung volumes, respectively, with no patient exceeding a relative lung volume difference of 0.2. Ultimately, 96% (24/25) of the patients achieved highly reproducible DIBH after coaching. In this study, the individualized determination of breath-hold thresholds was critical in ABC-assisted DIBH left breast radiotherapy. Through a standardized process, it objectively identified which patients required additional assistance and which were prepared to proceed. After coaching, the observed differences in lung volume between successive breath-holds were minimal, indicating excellent reproducibility of the chest anatomy positioning and enabling more precise left-sided breast radiotherapy.

**Supplementary Information:**

The online version contains supplementary material available at 10.1038/s41598-025-30484-6.

## Introduction

Radiotherapy remains a standard treatment for patients with breast cancer^1^. However, heart dose exposure represents a significant complicating factor that may adversely affect long-term survival and quality of life^2,3^. Dose reduction to the heart is therefore critical for minimizing complications. Notably, the increasing incidence of breast cancer among women has increased the importance of optimizing long-term outcomes^4^. Consequently, reducing the heart dose for patients with left-sided breast cancer has emerged as both clinically important and beneficial.

The deep inspiration breath-hold (DIBH) technique has been used to reduce the heart dose during radiotherapy through the modification of the chest anatomy^5–7^. By inducing lung volume expansion during inspiratory breath-hold, DIBH increases the distance between the chest wall and heart. However, this technique introduces potential uncertainties in breast radiotherapy, particularly regarding successive breath-hold chest anatomical variations ^8,9^. To optimize DIBH effectiveness, patients should maintain chest anatomical reproducibility across breath-holds while achieving maximal tolerable inspiratory capacity. Consequently, patient coaching becomes essential. Extended coaching regimens spanning several days or 1–2 weeks have demonstrated more benefits to patients in selected studies^10–13^. These findings collectively suggest that structured coaching enhances treatment efficacy through better DIBH.

The DIBH technique is currently recommended for left-sided breast radiotherapy^1^. However, standardized protocols and quantitative measurements for guiding the radiotherapy team in patient coaching are lacking^8,14,15^. Furthermore, existing DIBH-supporting technologies, including the active breathing coordinator (ABC), real-time position management (RPM), and optical surface monitoring system (OSMS), use fundamentally different operational principles^9,16,17^. Consequently, technique-specific coaching guidelines are scarce. In summary, despite being a recommended technique for left-sided breast radiotherapy, DIBH implementation lacks standardized coaching methodologies and objective assessment measurements.

This study presented a three-day DIBH coaching protocol using the ABC system (Elekta AB, Sweden), with particular emphasis on the breath-hold threshold and the lung volume difference from CT images. The former was a manually configured parameter in the ABC system that reflected the stability of patients’ respiratory maneuver during coaching. The latter was used to quantify the DIBH reproducibility. The aim of this protocol was to establish an objective coaching framework for radiation oncology teams, to optimize DIBH implementation in left-sided breast radiotherapy, and to improve reproducibility of chest anatomy.

## Methods

### Ethics approval and consent to participate

The institutional review board of the Wuhan No.1 Hospital approved this study. The study was conducted in accordance with the ethical standards of the institution, the 1964 Helsinki Declaration, and its later amendments or comparable ethical standards. Informed consent was obtained from all individual participants included in the study. No intervention was introduced in the actual treatments in this research, and patient identities were confidential.

### Patient information

This study enrolled 25 female patients with left-sided breast cancer who underwent radiotherapy at Wuhan No. 1 Hospital between December 2020 and March 2025 and participated in a three-day ABC-based coaching protocol. Eligible patients were required to be under 60 years of age, possess intact cognitive function, successfully pass a predefined breath-hold test (achieving three consecutive breath-holds of at least 30 s each), and voluntarily agreed to adopt DIBH during treatment. Ultimately, 24 patients successfully completed radiotherapy using the DIBH technique. One patient (#25) was transitioned to free-breathing (FB) radiotherapy due to air leakage and was consequently excluded from all subsequent analysis.

### Patient coaching protocol

**Fig. 1:**
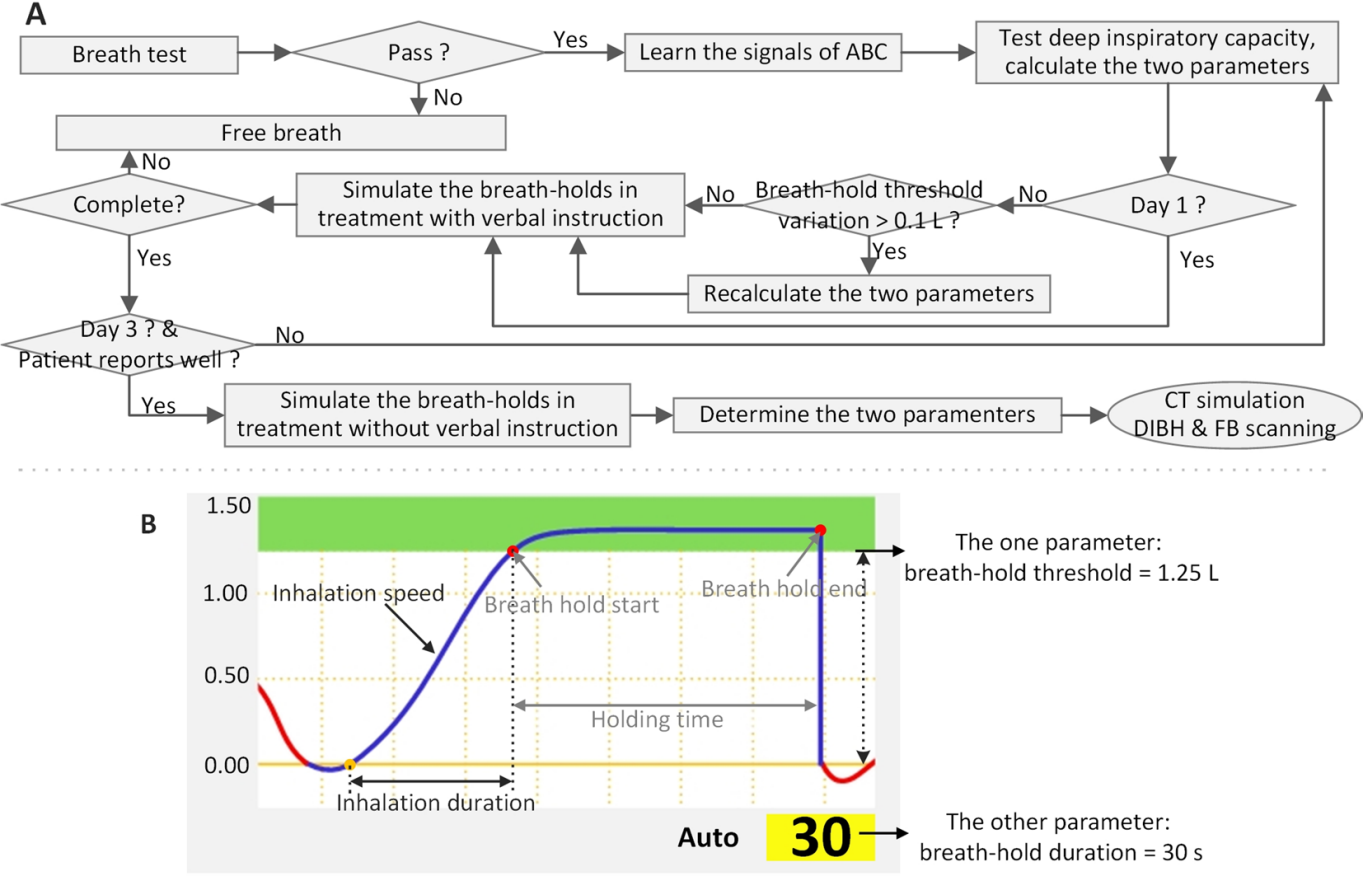
The ABC-based three-day coaching protocol. (**a**) Flowchart of the protocol. The breath-hold threshold was determined by analyzing maximal deep-inspiration volumes from 10 consecutive measurements at the beginning of each daily coaching session. (**b**) Representative respiratory curve during DIBH. The ABC system required individualized configuration of two device-specific parameters based on each patient’s respiratory performance. The breath-hold duration was initially set to 30 s (with an adjustment window), and inhalation duration and inhalation speed were calculated from the respiratory curves.

The ABC-based three-day coaching protocol was implemented over three consecutive days (Fig. 1a). On Day 1, eligible patients underwent initial screening through a standardized breath-hold test. Patients were positioned supine on the treatment table in the observation room and instructed to pinch their nose and breathe through their mouth. A staff recorded the maximum holding time during three attempts, with successful qualification defined as ≥ 30 s for all trials. Non-qualifying patients were either recommended home practice or transitioned to FB radiotherapy.

For qualified patients, the ABC system requires an individualized configuration of two device-specific parameters based on each patient’s respiratory performance (Fig. 1b): (1) the breath-hold threshold (automatically calculated as 70–80% of the mean maximal deep inspiration volume from 10 measurements) and (2) the breath-hold duration (initially set to 30 s, with an adjustment window). Following this configuration, patients were instructed to watch the screen and actively inspire until the respiratory curve entered the green zone. Patients then completed ABC familiarization coaching until consistent responses to visual cues and DIBH execution were demonstrated, which was verified through guided treatment simulation.

Day 2 focused on parameter optimization and skill reinforcement. The breath-hold duration was optimized based on the patient’s tolerance. Specifically, it was shortened if involuntary diaphragmatic motion was observed repeatedly, as this indicated that the patient had reached their limit. Regarding the breath-hold threshold, stability of this parameter was defined as a difference in the daily measured threshold of ≤ 0.1 L on two consecutive days. The coaching was continued until this stability criterion was met.

The final day comprised parameter confirmation and unassisted DIBH simulation. Patients underwent DIBH without verbal guidance to evaluate independent compliance. Those demonstrating difficulties received additional coaching sessions. Throughout the intervention, patients retained the option to transition to FB radiotherapy at any point.

All 25 patients completed a minimum of three days of coaching, with a subset requiring an additional practice day to achieve proficiency. The complete distribution of coaching durations across the cohort was detailed in Supplementary Table S1.

### Computed tomography (CT) simulation

All patients underwent CT simulation in the supine position with both arms elevated using customized vacuum immobilization. A total of three CT scans were performed for each DIBH patient (3 mm slice thickness). The first scan was acquired under FB conditions. The second scan was initiated at the beginning of one breath-hold, and the third scan was initiated 10–20 s after the start of another breath-hold. The two DIBH scans were acquired during two distinct breath-holds. Air leakage typically begins during the mid-phase of breath-holding.

These datasets served as the definitive evidence for treatment modality selection. The criterion for DIBH reproducibility was a markedly smaller difference in lung volume between the two DIBH scans than between the DIBH and FB scans. To visually confirm this quantitative assessment and directly demonstrate anatomical reproducibility, vertebral-based registration of sequential DIBH scans was performed. The resulting sternocostal overlap confirmed stable breath-hold maintenance, thereby validating the quantitative findings (Registered CT images of a typical patient are provided in Supplementary Figure S1).

Organ-at-risk contours were automatically generated using PV-med software (v1.0.0.19001; Perception Vision Medical Technologies). All structures underwent independent peer review using the Monaco treatment planning system (v6.00.11; Elekta) by a second radiation oncologist as part of the institutional quality assurance protocols.

### Data analysis

In this study, the reproducibility of DIBH was quantified by the difference in lung volume between the two DIBH scans. This measurement served as the criterion for DIBH suitability, which was defined as a markedly smaller difference in lung volume between the two DIBH scans than between the DIBH and FB scans (as described in the CT simulation). The relative difference in lung volume was normalized to the lung volume in the FB CT scan, using the formula: (DIBH volume–FB volume)/FB volume.

During the coaching process, three parameters were monitored to quantitatively assess patient respiratory maneuver: breath-hold threshold, inhalation duration, and inhalation speed (Fig. 1b and Supplementary Table S1). The stability of the breath-hold threshold was defined as a difference of ≤ 0.1 L in the daily measured value on two consecutive days. This stability criterion also served as the objective basis for determining the need for additional coaching (as described in the Patient Coaching Protocol).

Statistical analyses were conducted using MATLAB (R2022a, MathWorks, Inc.) with a significance threshold of *α* < 0.05 for all tests. The study incorporated both comparative analysis of means and correlation analysis. Normality was assessed using the Shapiro–Wilk test, with normally distributed datasets subsequently evaluated for homogeneity of variance using Levene’s test. When variance homogeneity was confirmed, between-group comparisons were performed using Student’s t test; otherwise, Welch’s t test was applied. For nonnormally distributed data, the Mann–Whitney U test was used for median comparisons. Correlation analyses used Pearson’s coefficient for normally distributed variables and Spearman’s rank correlation for nonnormally distributed variables.

## Results

### Dynamic changes in respiratory measurements during coaching sessions

**Fig. 2:**
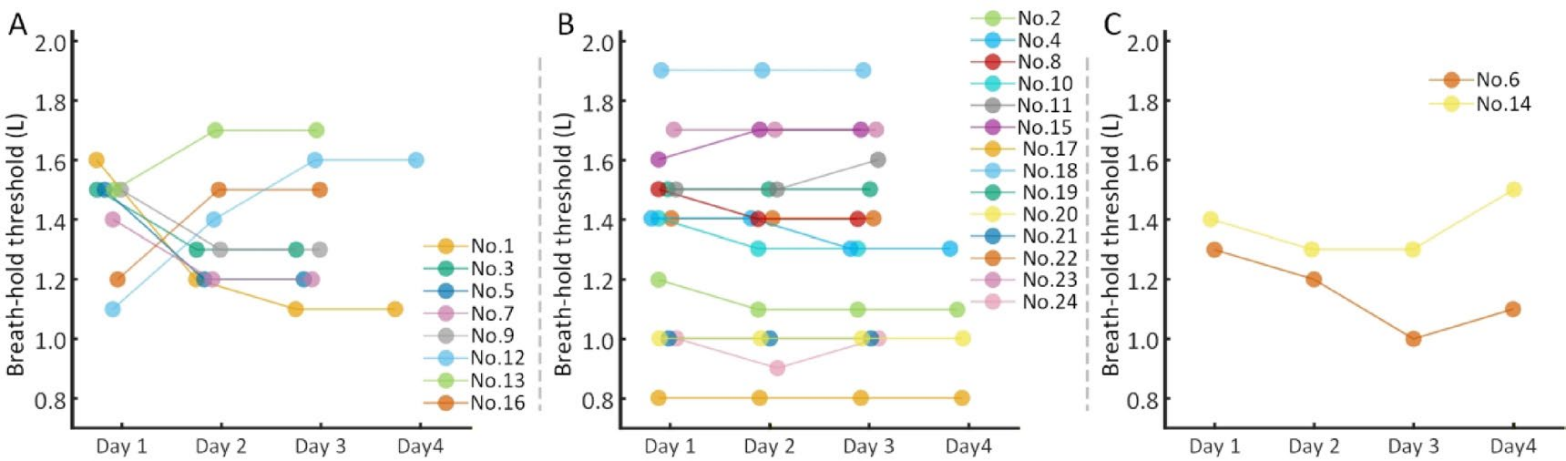
Dynamic changes in breath-hold threshold during coaching sessions. The stability of breath-hold threshold was defined as a day-to-day variation of ≤ 0.1 L between consecutive coaching sessions. Breath-hold threshold trajectories revealed four distinct patterns, including improvement, consistency, fluctuation and deterioration. (**a**) Eight patients presented improvement of breath-hold thresholds stability. These patients exhibited instability at the beginning of the coaching period but achieved stability by its conclusion. (**b**) Fourteen patients presented consistency of stability, who maintained a stable threshold throughout the entire coaching process. (**c**) The fluctuation pattern was observed in one patient (#6), who was initially stable, became unstable during the middle phase, and returned to stability by the final phase. The deterioration pattern was observed in one patient (#14). Overall, 41.7% (10/24) of the patients exhibited instability of the breath-hold threshold during the coaching period, and 9 of these 10 patients achieved stability by the final day. By the final coaching day, 95.8% (23/24) of patients achieved a stable breath-hold threshold.

The stability of the breath-hold threshold was defined as a difference in the daily measured threshold of ≤ 0.1 L on two consecutive days. Figure 2 shows the dynamic changes in the breath-hold threshold for each patient. Between coaching day 1 and day 2, 8 patients did not achieve a stable breath-hold threshold, including #1 (0.4 L difference), #3 (0.2 L), #5 (0.2 L), #7 (0.2 L), #9 (0.2 L), #12 (0.3 L), #13 (0.2 L), and #16 (0.3 L), whereas 16 patients achieved stability. Between day 2 and day 3, only 2 patients did not achieve stability, including #6 (0.2 L) and #12 (0.2 L), whereas 22 patients achieved it. Between day 3 and day 4, only patient #14 did not achieve stability (0.2 L). By this point, 8 patients had achieved stability. Fifteen patients, having achieved a stable breath-hold threshold by day 3, did not require coaching on day 4.

Overall, 41.7% (10/24) of the patients exhibited instability of the breath-hold threshold during the coaching period, and 9 of these 10 patients achieved stability by the final day. Patient #14 showed a 0.2 L difference in the breath-hold threshold between coaching day 3 and day 4 but declined further coaching sessions. Fourteen patients maintained stability throughout all coaching days. By the final coaching day, 95.8% (23/24) of patients achieved a stable breath-hold threshold.

Stability in both inhalation duration and speed was exhibited in eight patients (#8, #9, #10, #15, #16, #19, #20, #23). A notable overlap was observed, with 5 of these patients (#8, #15, #19, #20, #23) also showing stability in their breath-hold threshold. The remaining patients, however, generally exhibited fluctuations in these two metrics. Comparisons with Day 1 baseline values revealed that 58.3% (14/24) and 54.1% (13/24) of patients experienced statistically significant changes in inhalation duration and speed, respectively (*p* < 0.05).

Complete datasets, including individual breath-hold thresholds, inhalation speed, inhalation duration and statistical details, are provided in Supplementary Table S1.

### Breath-hold thresholds strongly correlated with DIBH-related lung volume

**Fig. 3:**
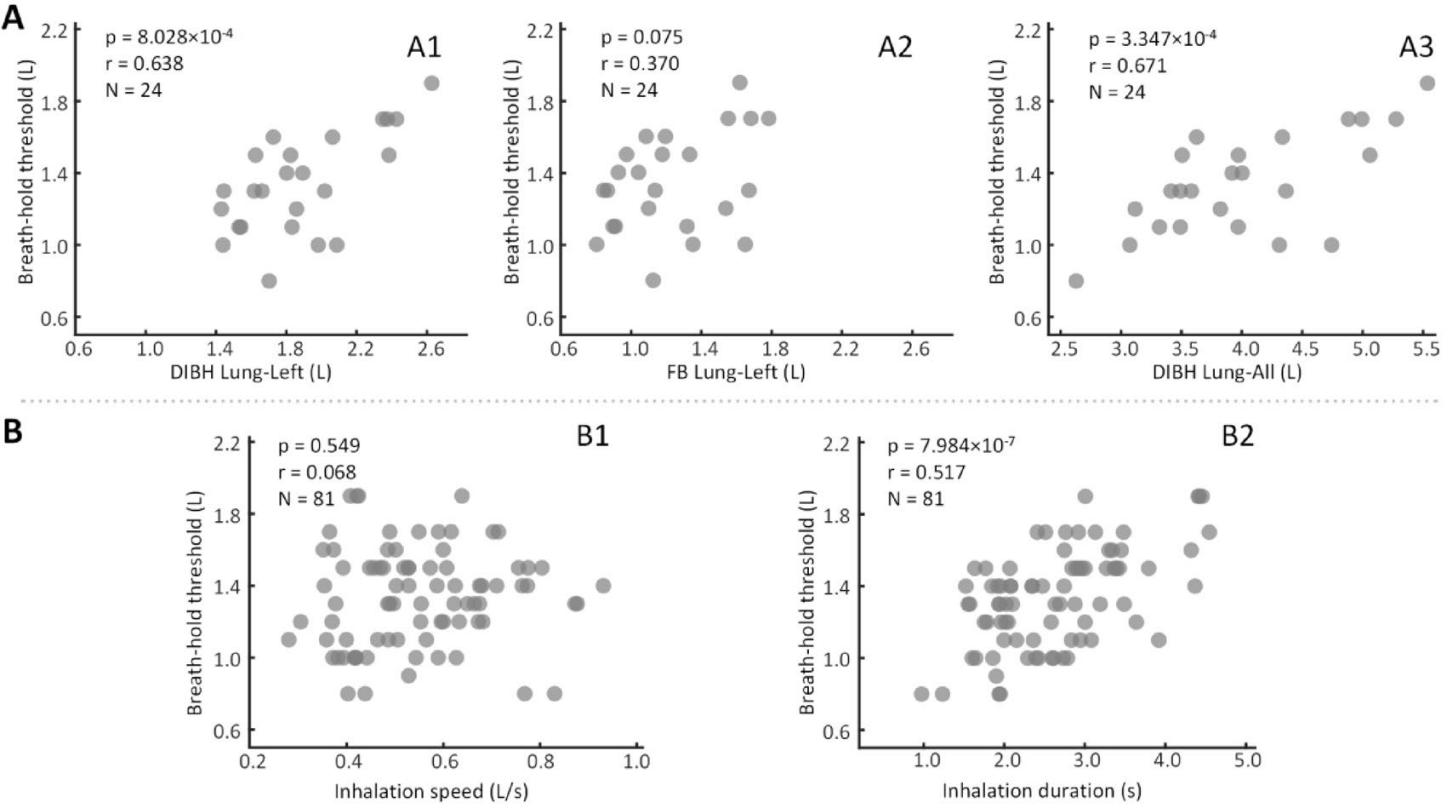
Correlation analysis of respiratory measurements. (**a**) The breath-hold threshold showed a significant positive correlation with DIBH left lung volume (**a1**) and total lung volume (**a3**), but no significant correlation with FB lung volume (**a2**). (**b**) The breath-hold threshold was positively correlated with inhalation duration (**b2**) but not with inhalation speed (**b1**).

The breath-hold threshold was significantly positively correlated with DIBH left lung volume (Fig. 3a, *r* = 0.638, *p* = 8.028 × 10^−4^), right lung volume (*r* = 0.647, *p* = 6.284 × 10^−4^), and total lung volume (*r* = 0.671, *p* = 3.347 × 10^−4^), whereas no significant correlations were observed with FB lung volume (left: *p* = 0.055; right: *p* = 0.055; total: *p* = 0.058). Regarding respiratory parameters, the breath-hold threshold showed positively correlated with inhalation duration (Fig. 3b, *r* = 0.517, *p* = 7.894 × 10^−^^7^), but was no significant association with inhalation speed (*p* = 0.549).

### The difference in the DIBH lung volume between breath-holds

**Fig. 4:**
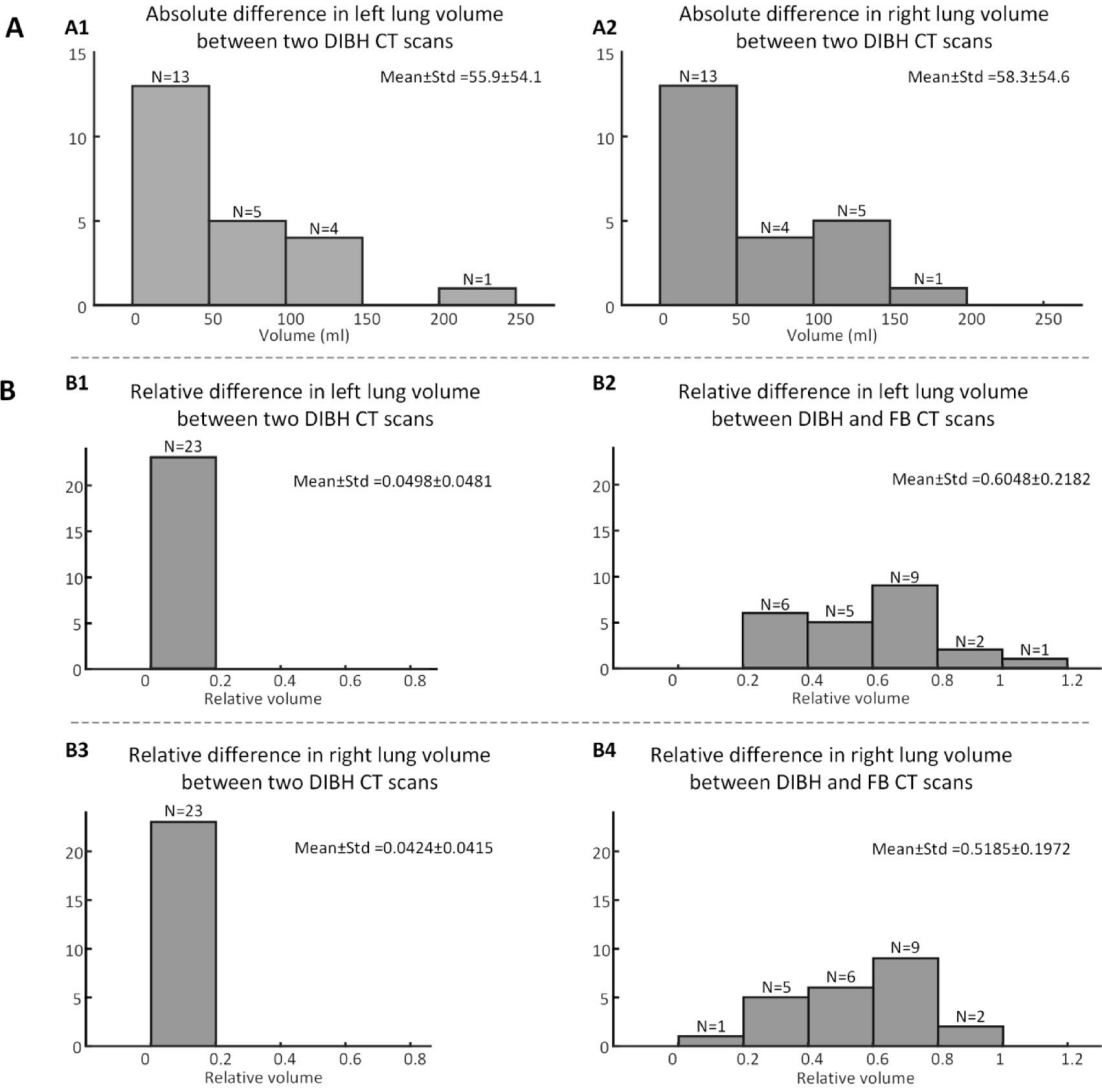
Quantitative assessment of chest anatomy reproducibility. (**a**) Histograms present left (**a1**) and right (**a2**) lung volume differences between two DIBH scans. (**b**) Histograms present the left (**b1**, **b2**) and right (**b3**, **b4**) lung volume differences between two DIBH scans and between DIBH and FB scans, respectively. Relative difference in lung volume was normalized by the lung volume in FB CT scan, i.e. (DIBH–FB)/FB. Y-axis represents the number of patients.

The difference of lung volume between breath-holds were analyzed in 23 patients (Fig. 4). Data of Patient 5 were excluded from the analysis due to CT scanning motion artifacts. Complete datasets, including individual lung volume of two DIBH scans and one FB scan, are provided in Supplementary Table S2 and S3.

For left lung volumes, 78.3% (18/23) of patients showed differences ≤ 0.1 L between two DIBH scans. Similar patterns were observed for right lung volumes, as 73.9% (17/23) of patients had differences ≤ 0.1 L between two DIBH scans. The relative lung volume differences were calculated with normalization to the FB volume. As shown in Fig. 4b, the difference for both the left and right lungs between the two DIBH scans was ≤ 0.2 for all patients, whereas the difference between DIBH and FB scans exceeded 0.2 in all but one case. Notably, the DIBH lung volume was significantly greater than the FB volume, with the left lung volume increasing from 1.230 ± 0.309 L (FB) to 1.885 ± 0.346 L (DIBH, *p* = 1.191 × 10^−^^8^) and the right lung volume increasing from 1.466 ± 0.404 L (FB) to 2.133 ± 0.444 L (DIBH, *p* = 1.957 × 10^−^^6^).

Statistical analysis revealed that the differences in the lung volume between the two DIBH scans did not follow a normal distribution (left *p* = 0.008, right *p* = 0.011). However, differences between DIBH and FB CT scans were normally distributed (left *p* = 0.668, right *p* = 0.918). All individual lung volume measurements from CT scans, whether DIBH or FB, followed normal distributions (DIBH left *p* = 0.168, right *p* = 0.061; FB left *p* = 0.132, right *p* = 0.542).

## Discussion

This study aimed to evaluate a three-day coaching protocol for DIBH that utilized quantitative optimization of the breath-hold threshold with an ABC device. Unlike RPM or SGRT systems that rely on surface imaging, the ABC system utilizes a balloon valve to directly control inhaled volume and enforce breath-hold once a preset threshold is reached^17^. Therefore, establishing a stable and personalized breath-hold threshold is crucial for treatment reproducibility. In this study, the breath-hold threshold was utilized as an objective process parameter to evaluate respiratory maneuver during coaching. For this purpose, its stability was defined as a day-to-day variation of ≤ 0.1 L between consecutive coaching sessions. Based on the stability criterion of the breath-hold threshold, data from the 24 patients revealed four distinct patterns of change: improvement, fluctuation, consistency, and deterioration.

The improvement group comprised 8 patients (33.3%). These patients exhibited instability at the beginning of the coaching period but achieved stability by its conclusion. The fluctuation pattern was observed in one patient (#6), who was initially stable, became unstable during the middle phase, and returned to stability by the final phase. The consistency group included 14 patients (58.3%), all of whom maintained a stable breath-hold threshold throughout the entire coaching process. The deterioration pattern was observed in one patient (#14). Although she had demonstrated a stable breath-hold threshold during the first three days of coaching, a change exceeding 0.1 L occurred on the fourth day, after which she declined further coaching sessions. It is noteworthy that the patient had self-reported air leakage during the initial three sessions. The cause of the leakage was identified and rectified on the fourth day. Therefore, only the data from the final day were considered an accurate reflection of her breath-hold capability, whereas the data from the first three days were invalid due to a measurement artifact.

Thus, a key contribution of this coaching protocol was that, through a standardized process, it objectively identified which patients required additional assistance and which were prepared to proceed. Ultimately, 95.8% (23/24) of the patients achieved stability prior to CT simulation.

Furthermore, a significant positive correlation was observed between the breath-hold threshold and DIBH lung volume. The significance of this finding was twofold. First, it suggested an association between the threshold and actual lung expansion during treatment, supporting its potential use as a process monitoring metric. Second, considering that larger DIBH volumes generally improve heart sparing^8,9^, the correlation indicated that maintaining a stable threshold could indirectly help optimize treatment efficacy.

In this study, patient eligibility for clinical treatment using the ABC device was determined based on DIBH reproducibility. Following coaching, each patient underwent three CT scans during simulation (two DIBH and one FB). These three sets of CT images were used to quantitatively assess eligibility for ABC-assisted treatment. The criterion for DIBH reproducibility was a markedly smaller difference in lung volume between the two DIBH scans than between the DIBH and FB scans. We designed this criterion considered two key factors. First, the high similarity in lung volume between the two DIBH scans indicated high DIBH reproducibility. Second, the significantly larger lung volume in DIBH scan compared to FB scan confirmed adequate lung expansion, which was fundamental for achieving the DIBH goal of heart protection.

Among the 25 patients, twenty-four met this criterion. The relative lung volume difference between the two DIBH scans was below 0.2 for all 24 patients, whereas the relative volume difference between DIBH and FB was predominantly greater than 0.2 (Fig. 4b). Only one patient exhibited a DIBH-to-FB relative volume difference below 0.2 in the right lung. CT images for this patient indicated pleural effusion, a condition that was a potential contributing factor to the impaired lung function.

Notably, Patient 14 also met the reproducibility criterion despite demonstrating a deteriorating pattern in her breath-hold threshold data. The absolute volume differences between her two DIBH scans were 21.9 mL (left) and 6.8 mL (right), corresponding to relative differences of 0.019 and 0.005, respectively. In comparison, the absolute differences between DIBH and FB volumes were 450.8 mL (left) and 538.9 mL (right), with relative differences of 0.384 and 0.402. These data indicate that Patient 14 achieved high DIBH reproducibility and a substantially larger lung volume during DIBH compared to FB.

Patient 5, whose data were excluded from the lung volume analysis (Fig. 4) due to motion artifacts in the CT scan, also met this criterion. Her FB scan during simulation had been affected by these artifacts. We identified his prior CT images acquired under FB conditions, which were used for comparison and reference. The data indicated that adequate lung expansion was achieved, and high reproducibility was observed between the two DIBH scans (Supplementary Table S1 and S2).

The only patient who did not meet the criterion after coaching was Patient 25. The absolute lung volume differences between the two DIBH scans were 308.3 mL (left) and 352.4 mL (right), corresponding to relative differences of 0.189 and 0.191. The absolute differences between DIBH and FB were 351.7 mL (left) and 462.6 mL (right), with relative differences of 0.189 and 0.250. These data revealed a considerable lung volume difference between the two DIBH scans, a finding that was also visually confirmed on CT images (see Supplementary Figure S2). Therefore, this patient was considered to have low DIBH reproducibility and was subsequently transferred to FB treatment. During clinical coaching, it was observed that the patient’s abdomen expanded upon inspiration but deflated approximately 20 s into the breath-hold. Although the patient did not self-report air leakage, this observation led us to speculate that undetected air leakage may have occurred and persisted throughout the coaching process.

This study used the difference in lung volume as the definitive evidence of the protocol’s effectiveness. The objective, image-based quantitative results demonstrated that 96% (24/25) of the patients achieved a highly reproducible chest anatomy after completing the coaching protocol, which established a solid foundation for the precise delivery of DIBH radiotherapy. Given this high reproducibility in respiratory control, these results also support the potential application of this coaching technique for other thoracic radiotherapy treatments where respiratory motion management, such as lung radiotherapy^18,19^, is crucial.

On the other hand, inhalation speed and duration showed no consistent trend, which might reflect the inherent complexity of respiratory patterns. Given that previous literature suggests anxiety can influence respiratory maneuver performance^20,21^ and that some patients in our study self-reported nervousness during coaching, we tentatively speculate that psychological factors could play a role in unstable inhalation metrics. However, this study did not incorporate quantitative psychological assessments to confirm this mechanism. Including such metrics in future research represents an important direction.

The three-day coaching protocol presented in this study placed relatively high demands on patient compliance and increased the clinical workload for the radiation team. These factors may limit the broader applicability of the protocol. One relevant finding was that 5 patients with stable respiratory patterns demonstrated consistency in both breath-hold thresholds and inhalation metrics. However, based on the available data, we were unable to predict which patients would not require coaching. These parameters may contain a preliminary hint that could point toward indicators of DIBH stability. Future studies should explore the predictive value of these parameters for early DIBH stability, with the goal of reducing the number of coaching sessions needed.

## Conclusion

This study proposes an ABC-based three-day coaching protocol for DIBH that utilizes the breath-hold threshold as a key process parameter and the lung volume difference from CT images as the definitive validation metric of DIBH reproducibility. The results demonstrated that for the 9 patients who exhibited fluctuations in their breath-hold threshold, continued coaching successfully enabled them to achieve stability. Ultimately, 96% (24/25) of the patients achieved highly reproducible DIBH after coaching. This reproducibility ensures a reliable foundation for the precise delivery of radiotherapy in left-sided breast cancer.

## Supplementary Information

Below is the link to the electronic supplementary material.

Supplementary Material 1

## Data Availability

The datasets used and/or analysed during the current study are available from the corresponding author on reasonable request.

## References

[1] Gradishar, W. J. et al. Breast cancer, Version 3.2024, NCCN clinical practice guidelines in oncology. *J. Natl. Compr. Cancer Netw.* **22**, 331–357. 10.6004/jnccn.2024.0035 (2024). 10.6004/jnccn.2024.0035 39019058

[2] Desai, N., Currey, A., Kelly, T. & Bergom, C. Nationwide trends in heart-sparing techniques utilized in radiation therapy for breast cancer. *Adv. Radiat. Oncol.* **4**, 246–252. 10.1016/j.adro.2019.01.001 (2019). 31011669 10.1016/j.adro.2019.01.001 PMC6460327

[3] Darby, S. C. et al. Radiation-Related Heart Disease: Current Knowledge and Future Prospects. *Int. J. Radiat. Oncol.*Biol.*Phys.* **76**, 656–665. 10.1016/j.ijrobp.2009.09.064 (2010). 20159360 10.1016/j.ijrobp.2009.09.064 PMC3910096

[4] Siegel, R. L., Kratzer, T. B., Giaquinto, A. N., Sung, H. & Jemal, A. Cancer statistics, 2025. *CA: Cancer J. Clin.* **75**, 10–45. 10.3322/caac.21871 (2025). 39817679 10.3322/caac.21871 PMC11745215

[5] Mayr, N. A. et al. Reducing cardiac radiation dose from breast cancer radiation therapy with breath hold training and cognitive behavioral therapy. *Top. Magn. Reson. Imaging* **29**, 135–148. 10.1097/RMR.0000000000000241 (2020). 32568976 10.1097/RMR.0000000000000241

[6] Chatterjee, S. et al. Resource requirements and reduction in cardiac mortality from deep inspiration breath hold (DIBH) radiation therapy for left sided breast cancer patients: A prospective service development analysis. *Pract. Radiat. Oncol.* **8**, 382–387. 10.1016/j.prro.2018.03.007 (2018). 29699893 10.1016/j.prro.2018.03.007

[7] Hoeller, U. et al. Late sequelae of radiotherapy. *Dtsch. Arztebl. Int.*10.3238/arztebl.m2021.0024 (2021). 34024324 10.3238/arztebl.m2021.0024 PMC8278127

[8] Latty, D., Stuart, K. E., Wang, W. & Ahern, V. Review of deep inspiration breath-hold techniques for the treatment of breast cancer. *J. Med. Radiat. Sci.* **62**, 74–81. 10.1002/jmrs.96 (2015). 26229670 10.1002/jmrs.96 PMC4364809

[9] Boda-Heggemann, J. et al. Deep inspiration breath hold—based radiation therapy: A clinical review. *Int. J. Radiat. Oncol.*Biol.*Phys.* **94**, 478–492. 10.1016/j.ijrobp.2015.11.049 (2016). 26867877 10.1016/j.ijrobp.2015.11.049

[10] Kefeli, A. U. et al. Patient coaching for deep inspiration breath hold decreases set-up duration and left anterior descending artery dose for left-sided breast cancer radiotherapy. *Support. Care Cancer*10.1007/s00520-025-09446-1 (2025). 40240656 10.1007/s00520-025-09446-1 PMC12003517

[11] Kim, A. et al. Effects of preparatory coaching and home practice for deep inspiration breath hold on cardiac dose for left breast radiation therapy. *Clin. Oncol.* **30**, 571–577. 10.1016/j.clon.2018.04.009 (2018). 10.1016/j.clon.2018.04.009 29773446

[12] Kalet, A. M. et al. The dosimetric benefit of in-advance respiratory training for deep inspiration breath holding is realized during daily treatment in left breast radiotherapy: A comparative retrospective study of serial surface motion tracking. *J. Med. Imaging Radiat. Oncol.* **65**, 354–364. 10.1111/1754-9485.13181 (2021). 33932102 10.1111/1754-9485.13181 PMC8252041

[13] Oonsiri, P., Wisetrinthong, M., Chitnok, M., Saksornchai, K. & Suriyapee, S. An effective patient training for deep inspiration breath hold technique of left-sided breast on computed tomography simulation procedure at King Chulalongkorn Memorial Hospital. *Radiat. Oncol. J.* **37**, 201–206. 10.3857/roj.2019.00290 (2019). 31591868 10.3857/roj.2019.00290 PMC6790791

[14] Bergom, C., Currey, A., Desai, N., Tai, A. & Strauss, J. B. Deep inspiration breath hold: Techniques and advantages for cardiac sparing during breast cancer irradiation. *Front. Oncol.*10.3389/fonc.2018.00087 (2018). 29670854 10.3389/fonc.2018.00087 PMC5893752

[15] Aznar, M. C. et al. ESTRO-ACROP guideline: Recommendations on implementation of breath-hold techniques in radiotherapy. *Radiother. Oncol.*10.1016/j.radonc.2023.109734 (2023). 37301263 10.1016/j.radonc.2023.109734

[16] Ball, H. J. et al. Results from the AAPM task group 324 respiratory motion management in radiation oncology survey. *J. Appl. Clin. Med. Phys.*10.1002/acm2.13810 (2022). 36316761 10.1002/acm2.13810 PMC9680579

[17] Keall, P. J. et al. The management of respiratory motion in radiation oncology report of AAPM Task Group 76a). *Med. Phys.* **33**, 3874–3900. 10.1118/1.2349696 (2006). 17089851 10.1118/1.2349696

[18] Tanaka, H. et al. Deep inspiration breath hold real-time tumor-tracking radiation therapy (DBRT) as a novel stereotactic body radiation therapy approach for lung tumors. *Sci. Rep.*10.1038/s41598-024-53020-4 (2024). 38287139 10.1038/s41598-024-53020-4 PMC10825222

[19] Lee, S. et al. Tumor localization accuracy for high-precision radiotherapy during active breath-hold. *Radiother. Oncol.* **137**, 145–152. 10.1016/j.radonc.2019.04.036 (2019). 31103912 10.1016/j.radonc.2019.04.036

[20] Meuret, A. E. & Ritz, T. Hyperventilation in panic disorder and asthma: Empirical evidence and clinical strategies. *Int. J. Psychophysiol.* **78**, 68–79. 10.1016/j.ijpsycho.2010.05.006 (2010). 20685222 10.1016/j.ijpsycho.2010.05.006 PMC2937087

[21] Dower, K., Halkett, G. K. B., Dhillon, H., Naehrig, D. & O’Connor, M. Eliciting the views of left breast cancer patients’ receiving deep inspiration breath hold radiation therapy to inform the design of multimedia education and improve patient-centred care for prospective patients. *J. Med. Radiat. Sci.* **71**, 384–395. 10.1002/jmrs.790 (2024). 38623813 10.1002/jmrs.790 PMC11569405

