# Supplementary material for: A three day coaching protocol for deep inspiration breath hold in left breast radiotherapy using active breathing control
Source: Sci Rep. 2025 Dec 4;15:43661. doi: 10.1038/s41598-025-30484-6 (PMC12701011; doi:10.1038/s41598-025-30484-6)
Supplement: Supplementary file 1 — Supplementary Material 1 [file 41598_2025_30484_MOESM1_ESM.pdf]

A three day coaching protocol for deep inspiration breath hold in left breast radiotherapy using active breathing control

Siqi Jiang[1], Haokun Shi[1], Xi Ye[1], Jun Yang[1], Jian Zheng[1], Peiwei Wu[1], Qiang Ye[1], Shuanglai Qing[1], Wenjie Liu[1], Zuowei Hu[1], Yu Cai[1*]

[1] Department of Radiation Oncology, Wuhan No.1 Hospital, Wuhan, Hubei, China

Correspondence author: Yu Cai

Email: caiyu2021@sina.com

Supplementary Table S1. The statistical analysis of coaching for each patient

Patient #	Coaching days	Breath-hold threshold (L)	Breath-holds N	Inhalation duration	p-value	Inhalation speed	p-value
1	day 1	1.6	12	3.45 ± 0.60	-	0.35 ± 0.06	-
	day 2	1.2	13	3.00 ± 0.45	**0.046**	0.37 ± 0.07	0.807
	day 3	1.1	20	2.36 ± 0.27	**4.255×10^{-5}**	0.46 ± 0.05	**4.117×10^{-6}**
	day 4	1.1	12	2.15 ± 0.14	**9.380×10^{-6}**	0.51 ± 0.03	**2.056×10^{-6}**
2	day 1	1.2	20	3.64 ± 0.68	-	0.30 ± 0.07	-
	day 2	1.1	12	3.92 ± 0.54	0.236	0.28 ± 0.04	0.150
	day 3	1.1	12	3.08 ± 0.45	**0.017**	0.36 ± 0.06	**0.028**
	day 4	1.1	19	2.83 ± 0.71	**4.204×10^{-4}**	0.40 ± 0.08	**4.685×10^{-4}**
3	day 1	1.5	18	3.36 ± 0.84	-	0.39 ± 0.09	-
	day 2	1.3	11	3.49 ± 0.64	0.436	0.38 ± 0.06	0.598
	day 3	1.3	7	2.88 ± 1.01	**0.045**	0.49 ± 0.15	0.053
4	day 1	1.4	11	1.52 ± 0.22	-	0.93 ± 0.13	-
	day 2	1.4	9	1.92 ± 0.33	**0.005**	0.71 ± 0.12	**0.001**
	day 3	1.3	10	2.02 ± 0.33	**6.760×10^{-4}**	0.65 ± 0.10	**2.626×10^{-5}**
	day 4	1.3	10	1.94 ± 0.15	**8.813×10^{-5}**	0.66 ± 0.05	**2.481×10^{-5}**
**5 Motion artifacts**	day 1	1.5	8	1.63 ± 0.38	-	0.76 ± 0.15	-
	day 2	1.2	14	1.78 ± 0.21	0.088	0.67 ± 0.08	**0.008**
	day 3	1.2	17	2.01 ± 0.23	0.098	0.60 ± 0.07	**0.008**
6	day 1	1.3	25	2.69 ± 0.82	-	0.49 ± 0.14	-
	day 2	1.2	18	1.96 ± 0.85	**1.734×10^{-4}**	0.55 ± 0.13	0.083
	day 3	1.0	11	2.78 ± 0.73	0.631	0.42 ± 0.12	0.154
	day 4	1.1	8	2.00 ± 0.43	**0.027**	0.56 ± 0.12	0.155
7	day 1	1.4	17	2.34 ± 0.43	-	0.53 ± 0.09	-
	day 2	1.2	14	1.75 ± 0.16	**3.763×10^{-5}**	0.68 ± 0.06	**7.631×10^{-6}**
	day 3	1.2	16	2.05 ± 0.41	0.054	0.60 ± 0.11	0.054
8	day 1	1.5	12	2.92 ± 1.61	-	0.61 ± 0.36	-
	day 2	1.4	11	2.47 ± 1.01	0.537	0.62 ± 0.19	0.885
	day 3	1.4	13	1.95 ± 0.70	0.086	0.77 ± 0.20	0.159
9	day 1	1.5	13	1.77 ± 0.40	-	0.80 ± 0.25	-
	day 2	1.3	14	1.57 ± 0.45	0.198	0.88 ± 0.21	0.396
	day 3	1.3	13	1.55 ± 0.41	0.124	0.87 ± 0.19	0.259
10	day 1	1.4	14	2.35 ± 0.62	-	0.59 ± 0.15	-

	day 2	1.3	13	2.63	±	1.13	0.922	0.55	±	0.19	0.618
	day 3	1.3	10	2.10	±	0.28	0.429	0.62	±	0.07	0.558
11	day 1	1.5	10	2.07	±	0.59	-	0.78	±	0.25	-
	day 2	1.5	13	2.84	±	0.51	**0.002**	0.57	±	0.09	**0.006**
	day 3	1.6	14	2.75	±	0.56	**0.008**	0.60	±	0.12	**0.033**
12	day 1	1.1	22	2.95	±	0.84	-	0.49	±	0.15	-
	day 2	1.4	14	4.37	±	0.57	**3.335×10^{-6}**	0.35	±	0.07	**0.004**
	day 3	1.6	15	4.32	±	0.67	**1.400×10^{-4}**	0.37	±	0.08	**0.008**
	day 4	1.6	10	3.30	±	0.35	0.215	0.48	±	0.05	0.967
13	day 1	1.5	14	3.79	±	0.52	-	0.45	±	0.06	-
	day 2	1.7	13	4.54	±	0.41	**3.182×10^{-4}**	0.36	±	0.05	**4.210×10^{-4}**
	day 3	1.7	10	3.48	±	0.37	0.117	0.49	±	0.05	0.084
**14**	day 1	1.4	14	2.74	±	0.79	-	0.50	±	0.13	-
	day 2	1.3	10	1.93	±	0.20	**0.001**	0.67	±	0.07	**8.294×10^{-4}**
	day 3	1.3	14	3.19	±	0.91	0.148	0.50	±	0.15	0.913
	day 4	1.5	16	2.96	±	0.53	0.119	0.52	±	0.11	0.884
15	day 1	1.6	11	3.34	±	0.76	-	0.50	±	0.15	-
	day 2	1.7	11	3.13	±	0.46	0.448	0.55	±	0.09	0.380
	day 3	1.7	7	2.92	±	0.46	0.212	0.59	±	0.10	0.193
16	day 1	1.2	10	2.58	±	0.82	-	0.63	±	0.21	-
	day 2	1.5	7	3.39	±	1.17	0.112	0.48	±	0.16	0.113
	day 3	1.5	7	3.43	±	0.98	0.073	0.47	±	0.16	0.101
17	day 1	0.8	30	1.95	±	0.54	-	0.40	±	0.13	-
	day 2	0.8	14	0.97	±	0.18	**6.877×10^{-7}**	0.83	±	0.14	**3.828×10^{-7}**
	day 3	0.8	20	1.23	±	0.60	**2.482×10^{-4}**	0.77	±	0.30	**5.126×10^{-5}**
	day 4	0.8	13	1.94	±	0.26	0.918	0.44	±	0.06	0.362
18	day 1	1.9	10	4.46	±	0.64	-	0.42	±	0.08	-
	day 2	1.9	9	4.41	±	0.54	0.702	0.42	±	0.08	0.918
	day 3	1.9	9	4.42	±	0.57	0.823	0.41	±	0.09	0.673
	day 4	1.9	10	3.01	±	0.46	**4.288×10^{-4}**	0.64	±	0.08	**1.237×10^{-5}**
19	day 1	1.5	19	3.00	±	1.08	-	0.53	±	0.22	-
	day 2	1.5	11	3.27	±	0.29	0.319	0.46	±	0.04	0.966
	day 3	1.5	13	2.89	±	0.49	0.734	0.53	±	0.09	0.179
20	day 1	1.0	9	2.61	±	0.34	-	0.38	±	0.05	-
	day 2	1.0	24	2.42	±	0.44	0.245	0.42	±	0.08	0.203
	day 3	1.0	16	2.59	±	0.50	0.918	0.39	±	0.08	0.700
	day 4	1.0	12	2.39	±	0.32	0.137	0.42	±	0.06	0.145
21	day 1	1.0	6	2.73	±	0.55	-	0.37	±	0.07	-
	day 2	1.0	14	2.29	±	0.40	0.058	0.44	±	0.08	0.074
	day 3	1.0	7	1.86	±	0.34	**0.005**	0.54	±	0.09	**0.002**
**22**	day 1	1.4	10	1.85	±	0.26	-	0.76	±	0.10	-
**sample**	day 2	1.4	10	2.08	±	0.31	0.087	0.68	±	0.09	0.064
**Fig.2A**	day 3	1.4	10	2.08	±	0.26	0.060	0.67	±	0.08	**0.034**
23	day 1	1.7	10	2.51	±	0.59	-	0.70	±	0.16	-
	day 2	1.7	14	2.76	±	0.30	0.238	0.62	±	0.06	0.126
	day 3	1.7	14	2.41	±	0.35	0.614	0.71	±	0.11	0.859
24	day 1	1.0	27	1.64	±	0.64	-	0.59	±	0.15	-
	day 2	0.9	17	1.90	±	0.29	**0.001**	0.53	±	0.08	0.087

	day 3	1.0	12	1.60	±	0.21	0.465	0.63	±	0.09	0.420
**25**	day 1	1.5	17	1.63	±	0.27	-	0.65	±	0.10	-
**Air**	day 2	1.1	18	2.87	±	0.88	**6.877×10^{-7}**	0.45	±	0.14	**4.075×10^{-5}**
**leakage**	day 3	1.2	15	2.28	±	0.71	**2.141×10^{-4}**	0.50	±	0.10	**0.001**
	day 4	1.1	12	2.29	±	0.52	**8.862×10^{-5}**	0.49	±	0.09	**2.584×10^{-4}**
	day 5	1.1	16	2.09	±	0.22	**7.748×10^{-6}**	0.52	±	0.05	**2.301×10^{-4}**

For each patient, all metric values were compared with those on day 1 values.

Supplementary Table S2. The left lung volume of each patient from CT simulation images

#	Breath-hold threshold (L)	Left Lung (ml)						
		DIBH A	DIBH B	FB	Difference A-B	Difference abs((A-B)/FB)	Difference A-FB	Difference abs((A-FB)/FB)
1	1.10	1832.418	1804.238	1315.157	28.180	0.021	517.261	0.393
2	1.10	1541.213	1633.658	907.214	-92.445	0.102	633.999	0.699
3	1.30	2018.722	2027.669	1667.901	-8.947	0.005	350.821	0.210
4	1.30	1661.917	1800.624	862.404	-138.707	0.161	799.513	0.927
5	1.20	1430.392	1398.997	1536.121	31.395	0.020	-105.729	0.069
6	1.10	1531.142	1534.061	895.651	-2.919	0.003	635.491	0.710
7	1.20	1858.277	1860.53	1096.465	-2.253	0.002	761.812	0.695
8	1.40	1801.643	1835.663	1039.476	-34.020	0.033	762.167	0.733
9	1.30	1444.294	1498.881	840.211	-54.587	0.065	604.083	0.719
10	1.30	1617.266	1505.823	1133.726	111.443	0.098	483.540	0.427
11	1.60	1726.032	1836.469	1082.704	-110.437	0.102	643.328	0.594
12	1.60	2063.327	2131.784	1192.182	-68.457	0.057	871.145	0.731
13	1.70	2374.542	2366.922	1549.587	7.620	0.005	824.955	0.532
14	1.50	1625.271	1603.429	1174.537	21.842	0.019	450.734	0.384
15	1.70	2349.269	2400.29	1678.718	-51.021	0.030	670.551	0.399
16	1.50	1823.688	1792.705	971.088	30.983	0.032	852.600	0.878
17	0.80	1702.397	1687.947	1122.526	14.450	0.013	579.871	0.517
18	1.90	2627.624	2638.739	1616.049	-11.115	0.007	1011.575	0.626
19	1.50	2383.967	2175.608	1330.097	208.359	0.157	1053.870	0.792
20	1.00	1439.46	1484.392	800.946	-44.932	0.056	638.514	0.797
21	1.00	2087.409	2174.34	1646.617	-86.931	0.053	440.792	0.268
22	1.40	1893.164	1918.831	924.58	-25.667	0.028	968.584	1.048
23	1.70	2426.791	2423.03	1779.787	3.761	0.002	647.004	0.364
24	1.00	1981.529	2107.597	1348.962	-126.068	0.093	632.567	0.469
25	1.10	1986.496	1678.165	1634.752	308.331	0.189	351.744	0.215

DIBH A denotes the first DIBH CT scan during CT simulation, DIBH B the second DIBH scan, and FB the free-breathing CT simulation. We took the absolute value (abs) of all negative measurements to ensure positive values.

The FB CT images of Patient 5 exhibited motion artifacts and were therefore excluded from lung volume comparisons. Consequently, we utilized a prior FB CT scan from this patient for reference. The DIBH lung volume was greater than FB volumes. The prior FB CT images showed that the volume of the left lung was 1106.3 mL.

Patients 14 reported air leakage during coaching, which was corrected prior to CT simulation, subsequently underwent DIBH for radiotherapy. In contrast, Patient 25 exhibited air leakage detected by calculation of difference in the lung volumes and was transitioned to FB radiotherapy after CT simulation.

Supplementary Table S3. The right lung volume of each patient from CT simulation images

#	Breath-hold threshold (L)	Right Lung (ml)						
		DIBH A	DIBH B	FB	Difference A-B	Difference abs((A-B)/FB)	Difference A-FB	Difference abs((A-FB)/FB)
1	1.10	2137.344	2103.439	1574.459	33.905	0.022	517.261	0.358
2	1.10	1949.685	2058.025	1164.608	-108.340	0.093	633.999	0.674
3	1.30	2346.193	2361.479	2023.575	-15.286	0.008	350.821	0.159
4	1.30	1829.161	1971.704	973.061	-142.543	0.146	799.513	0.880
5	1.20	1686.422	1626.589	1867.445	59.833	0.032	-105.729	0.097
6	1.10	1787.006	1784.153	1066.127	2.853	0.003	635.491	0.676
7	1.20	1964.62	1976.251	1304.106	-11.631	0.009	761.812	0.506
8	1.40	2118.659	2070.94	1276.707	47.719	0.037	762.167	0.659
9	1.30	1970.211	1973.514	1181.612	-3.303	0.003	604.083	0.667
10	1.30	1964.738	1844.427	1381.683	120.311	0.087	483.540	0.422
11	1.60	1899.458	2029.213	1348.024	-129.755	0.096	643.328	0.409
12	1.60	2272.691	2342.887	1396.324	-70.196	0.050	871.145	0.628
13	1.70	2507.437	2571.066	1657.504	-63.629	0.038	824.955	0.513
14	1.50	1880.942	1887.747	1342.039	-6.805	0.005	450.734	0.402
15	1.70	2643.023	2687.424	1962.297	-44.401	0.023	670.551	0.347
16	1.50	2147.91	2117.355	1192.83	30.555	0.026	852.600	0.801
17	0.80	926.086	919.545	722.304	6.541	0.009	579.871	0.282
18	1.90	2909.193	2899.548	1799.533	9.645	0.005	1011.575	0.617
19	1.50	2676.123	2480.512	1644.91	195.611	0.119	1053.870	0.627
20	1.00	1633.954	1687.62	967.326	-53.666	0.055	638.514	0.689
21	1.00	2655.608	2747.952	2175.718	-92.344	0.042	440.792	0.221
22	1.40	2109.145	2136.466	1226.012	-27.321	0.022	968.584	0.720
23	1.70	2850.113	2844.445	2304.539	5.668	0.002	647.004	0.237
24	1.00	2328.925	2447.306	1625.571	-118.381	0.073	632.567	0.433
25	1.10	2310.771	1958.428	1848.195	352.343	0.191	351.744	0.250

DIBH A denotes the first DIBH CT scan during CT simulation, DIBH B the second DIBH scan, and FB the free-breathing CT simulation. We took the absolute value (abs) of all negative measurements to ensure positive values.

The FB CT images of Patient 5 exhibited motion artifacts and were therefore excluded from right volume comparisons. Consequently, we utilized a prior FB CT scan from this patient for reference. The DIBH lung volume was greater than FB volumes. The prior FB CT images showed that the volume of the left lung was 1393.8 mL.

Patients 14 reported air leakage during coaching, which was corrected prior to CT simulation, subsequently underwent DIBH for radiotherapy. In contrast, Patient 25 exhibited air leakage detected by calculation of difference in the lung volumes and was transitioned to FB radiotherapy after CT simulation.

Supplementary Figure S1. The daily inhalation metrics and CT images of Patient 22

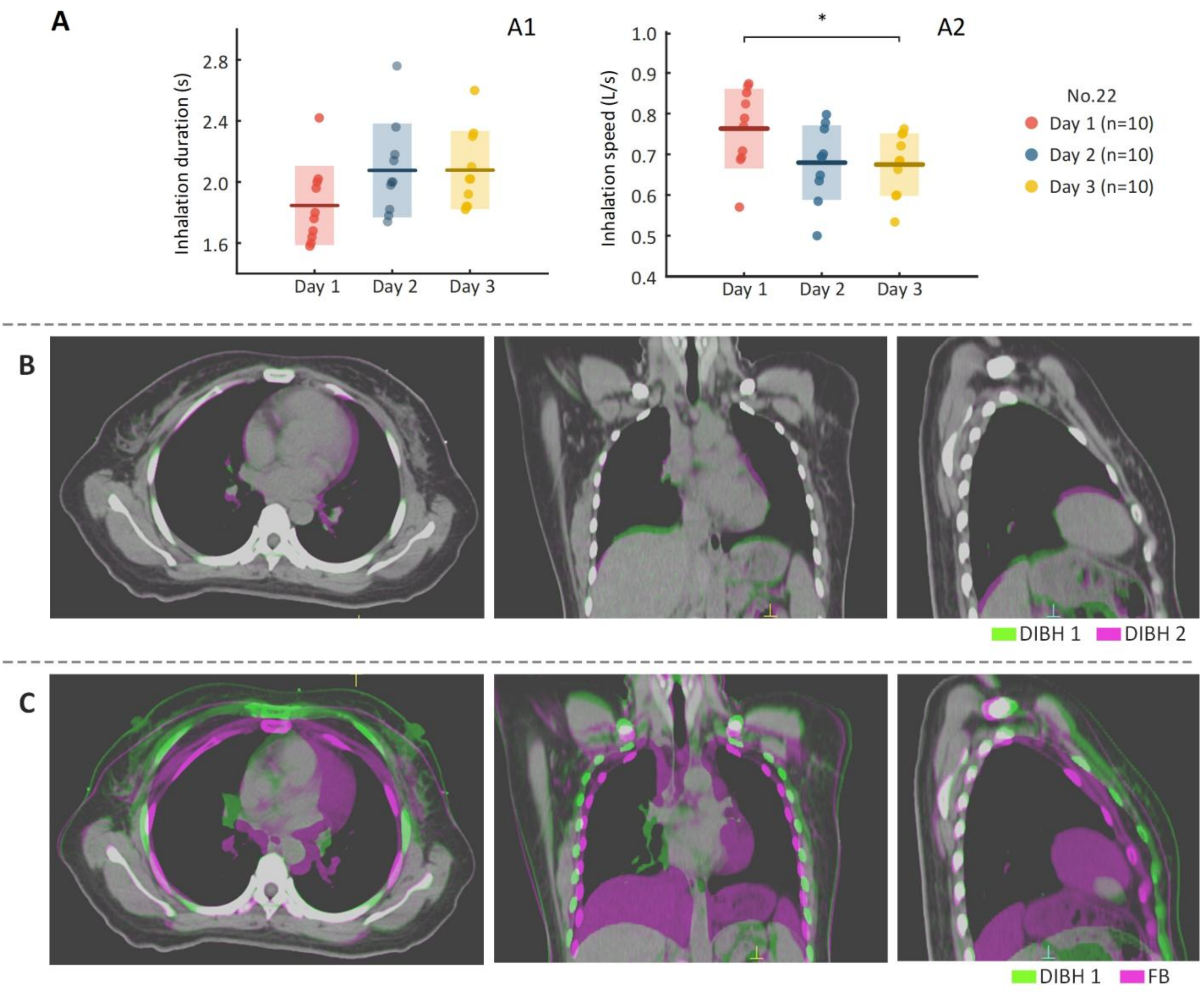

(a) Daily coaching data of inhalation duration (a1) and inhalation speed (a2) from Patient 22, showing 10 breath-holding measurements per day over three coaching days. Scatter points represent individual measurements each day, with horizontal lines and shaded areas indicating mean ± standard deviation. Inhalation speed values on Day 3 were significantly different from those on Day 1 (p = 0.0378). The quantitative analysis indicated that the Day 3 values of inhalation speed (0.67 ± 0.08 L/s, p = 0.0378) were significantly different from the Day 1 values (0.76 ± 0.10 L/s), whereas the Day 2 values (0.68 ± 0.09 L/s, p = 0.064) were not significantly different.

(b) Comparison of chest anatomy between two DIBH scans in Patient 22 shows near-complete anatomical overlap, with only minor heart position differences due to cardiac motion. Measured left and right lung volume differences were 25.67 mL and 26.32 mL, respectively.

(c) Comparison between DIBH and FB scans in Patient 22 shows distinct displacements in heart and liver positions, with left and right lung volume differences of 968.58 mL and 883.13 mL, respectively.

A comparison of the overlap patterns revealed distinct liver position differences between the DIBH and FB scans. In contrast, the two DIBH scans show nearly complete anatomical overlaps, with only minor heart position variations attributable to cardiac motion. These findings demonstrate excellent consistency with the differences in lung volume.

Supplementary Figure S2. The CT images of Patient 25

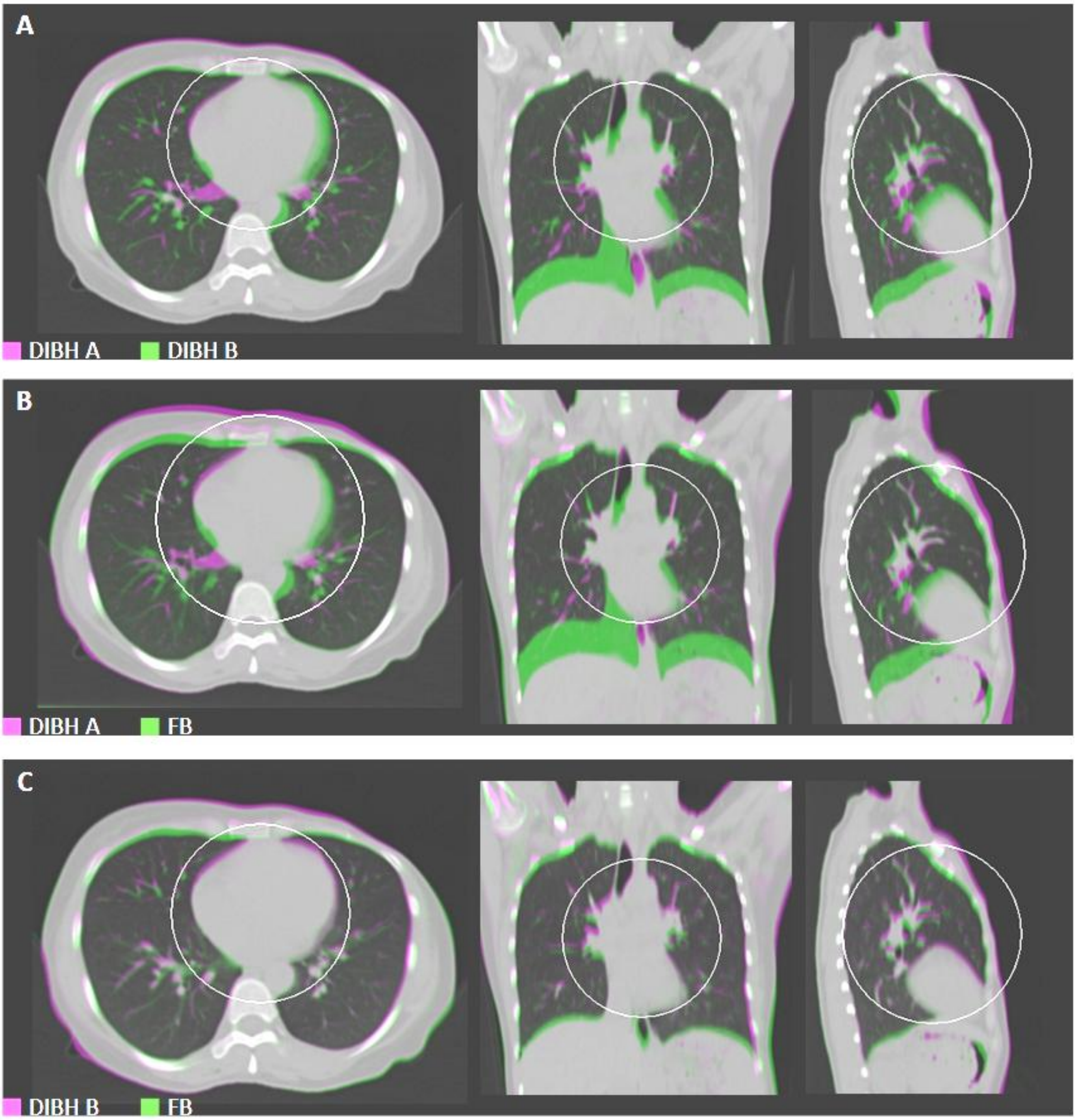

The analysis of CT images revealed significant air leakage, with left lung volumes measuring 1.986 L and 1.678 L in the two DIBH scans compared with 1.635 L in the FB scan. The lung volume difference (0.308 L) between the two DIBH scans approximated the DIBH-FB difference (0.351 L). Notably, the second DIBH scan showed better anatomical alignment with the FB scan than did the first DIBH scan. The values of lung volume were in the Supplementary Table S2 and S3.
